# Epigenetic roles in clonal hematopoiesis and aging kidney-related chronic kidney disease

**DOI:** 10.3389/fcell.2023.1281850

**Published:** 2023-10-20

**Authors:** Yoshiyasu Ogura, Imari Mimura

**Affiliations:** Division of Nephrology and Endocrinology, The University of Tokyo Graduate School of Medicine, Tokyo, Japan

**Keywords:** CHIP, aging, CKD, epigenetics, Klotho

## Abstract

Accumulation of somatic hematopoietic stem cell mutations with aging has been revealed by the recent genome-wide analysis. Clonal expansion, known as clonal hematopoiesis of indeterminate potential (CHIP), is a premalignant condition of hematological cancers. It is defined as the absence of definitive morphological evidence of a hematological neoplasm and occurrence of ≥2% of mutant allele fraction in the peripheral blood. In CHIP, the most frequently mutated genes are epigenetic regulators such as *DNMT3A, TET2,* and *ASXL1.* CHIP induces inflammation. CHIP is shown to be associated with not only hematological malignancy but also non-malignant disorders such as atherosclerosis, cardiovascular diseases and chronic liver disease. In addition, recent several large clinical trials have shown that CHIP is also the risk factor for developing chronic kidney disease (CKD). In this review article, we proposed novel findings about CHIP and CHIP related kidney disease based on the recent basic and clinical research. The possible mechanism of the kidney injury in CHIP is supposed to be due to the clonal expansion in both myeloid and lymphoid cell lines. In myeloid cell lines, the mutated macrophages increase the inflammatory cytokine level and induce chronic inflammation. It leads to epigenetic downregulation of kidney and macrophage klotho level. In lymphoid cell lines, CHIP might be related to monoclonal gammopathy of renal significance (MGRS). It describes any B cell or plasma cell clonal disorder that does not fulfill the criteria for cancer yet produces a nephrotoxic monoclonal immunoglobulin that leads to kidney injury or disease. MGRS causes M-protein related nephropathy frequently observed among aged CKD patients. It is important to consider the CHIP-related complications such as hematological malignancy, cardiovascular diseases and metabolic disorders in managing the elderly CKD patients. There are no established therapies for CHIP and CHIP-related CKD yet. However, recent studies have supported the development of effective CHIP therapies, such as blocking the expansion of aberrant HSCs and inhibiting chronic inflammation. In addition, drugs targeting the epigenetic regulation of Klotho in the kidney and macrophages might be therapeutic targets of CHIP in the kidney.

## 1 Introduction

Clonal hematopoiesis of indeterminate potential (CHIP) was originally proposed in the field of hematology. Recent genome-wide analyses have revealed that accumulation of somatic hematopoietic stem cell mutations and clonal expansion affect healthy populations. Clonal expansion is considered a pre-malignant condition in myelodysplastic syndrome (MDS) and acute myeloid leukemia (AML) (Genovese et al., 2014; Steensma et al., 2015; Miles et al., 2020).

Somatic mutations are the most common single-nucleotide variants (SNVs), small insertions or deletions, and copy number changes in large chromosomal regions (Jaiswal, 2020). Hematopoietic stem cells (HSCs) are estimated to acquire approximately 20 somatic mutations per year in their whole genome, most of which are SNVs (Osorio et al., 2018). Within the bone marrow, only long-lived HSCs can self-renew during an organism’s lifetime (Reya et al., 2001). Therefore, only mutations in the long-lived HSCs persist throughout the lifetime of an individual. The size of HSCs increases steadily in early life, reaching a stable plateau by adolescence. The estimated number of HSCs is 50,000–200,000 per person (Lee-Six et al., 2018). Humans are expected to harbor between 350,000 and 1,400,000 coding mutations within the HSC pool by 70 years of age (Jaiswal, 2020). If only one of these mutations is capable of providing a selective advantage to the HSC in which it arises, clonal expansion in the blood becomes prevalent during aging (Jaiswal and Ebert, 2019).

CHIP is different from hematological cancers and is defined by the absence of definitive morphological evidence of a hematological neoplasm and occurrence of ≥2% of mutant allele fraction in the peripheral blood (Steensma et al., 2015). The most commonly mutated genes in clonal hematopoiesis (CH) are *DNMT3A*, *TET2*, *ASXL1*, *PPM1D*, *JAK2*, *TP53*, and *SF3B1,* which are also commonly mutated in AML, MDS, and myeloproliferative neoplasms (Jaiswal, 2020). Loss-of-function mutations in two DNA methylation enzymes, *DNMT3A* and *TET2*, accounted for nearly two-thirds of CHIP cases. The third most commonly mutated gene is *ASXL1*, a chromatin regulator. The epigenetic regulators, *DNMT3A*, *TET2* and *ASXL1*, are essential for maintaining proper gene expression of HSC regulatory genes. Mutations of these genes cause the aberrant proliferation of HSCs (Challen et al., 2011; Solary et al., 2014; Fujino and Kitamura, 2020) *TP53* and *PPM1D* are DNA damage responsive genes. *JAK2* is involved in cellular growth signaling and *SF3B1* is a splicing factor (Jaiswal and Ebert, 2019). Mutations in CHIP can also be detected in circulating immune cells, such as granulocytes, monocytes, and lymphocytes (Jaiswal, 2020). This finding suggests that CHIP may lead to altered immune responses and non-malignant diseases associated with aging.

**FIGURE 1:**
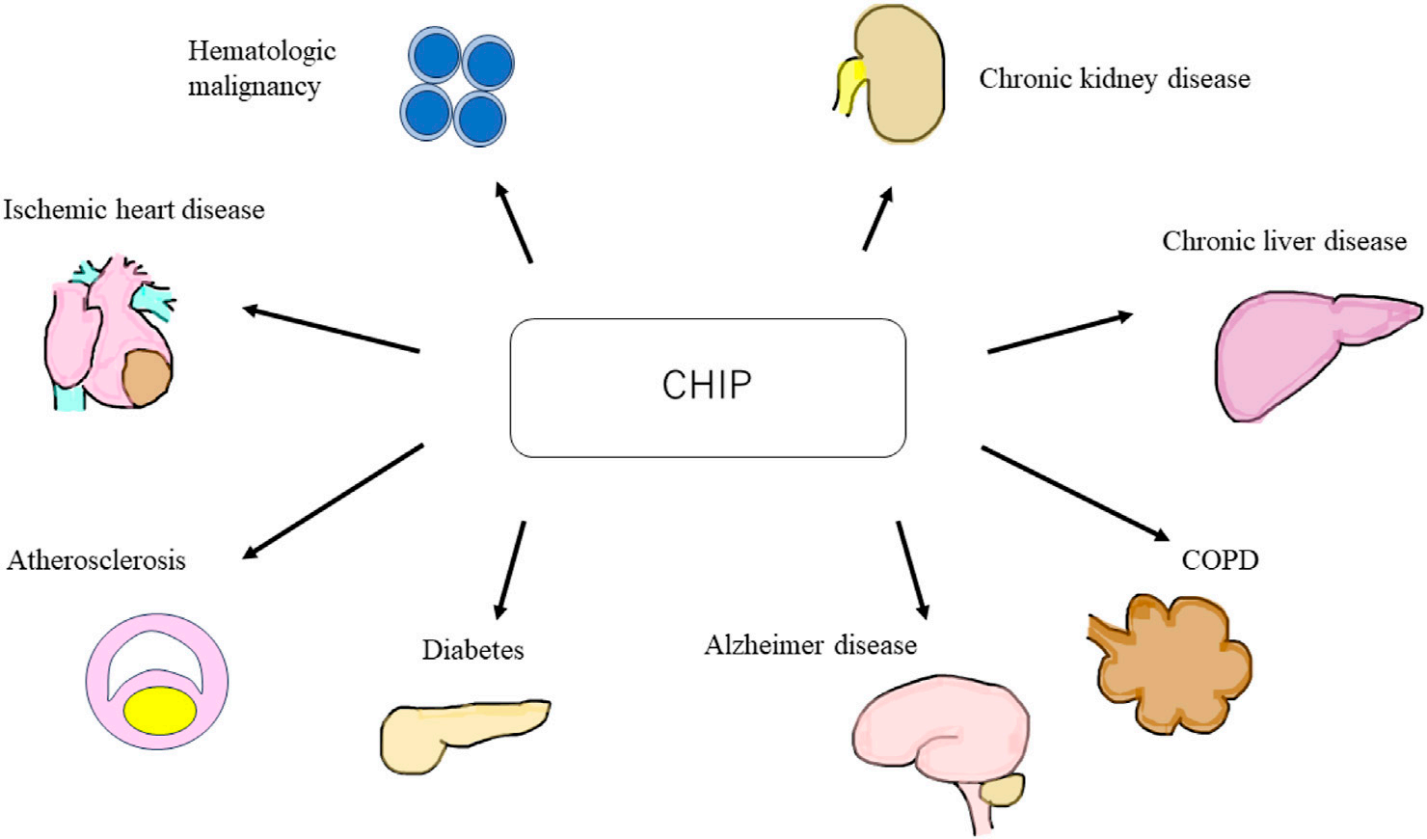
Schematic of clonal hematopoiesis of indeterminate potential (CHIP) related diseases. CHIP is a cause of hematologic malignant and non-malignant disorders, such as ischemic heart disease, atherosclerosis, diabetes, Alzheimer’s disease, chronic obstructive pulmonary disease, chronic liver disease, and chronic kidney disease. CHIP, clonal hematopoiesis of indeterminate potential; COPD, chronic obstructive pulmonary disease.

CHIP is an age-related disorder more commonly observed in older adults. Mutated HSCs and macrophages elevate inflammatory cytokine levels, leading to chronic inflammation (Jaiswal et al., 2014). A study showed that CHIP is associated with atherosclerotic cardiovascular disease (Jaiswal et al., 2017). Moreover, recent studies have shown that CHIP is a risk factor for non-malignant diseases, such as venous thrombosis (Hinds et al., 2016), type 2 diabetes (T2DM) (Fuster et al., 2020), age-related neurodegenerative diseases, such as Alzheimer’s disease (Bouzid et al., 2023), chronic obstructive pulmonary disease (COPD) (Buscarlet et al., 2017), and chronic liver disease (Jaiswal, 2020; Wong et al., 2023). Furthermore, CHIP has garnered attention as a few recent large clinical trials have reported that CHIP is a risk factor for chronic kidney disease (CKD) (Vlasschaert et al., 2022; Kestenbaum et al., 2023) (Figure 1).

## 2 Risk factors for inducing CHIP

Aging is the strongest risk factor of CHIP. Detectable somatic mutations are rare in persons <40 years but rise in frequency with age (Jaiswal et al., 2014). It has been reported that 10% of persons >65 years and the majority (62%) of individuals ≥80 years had CHIP (Genovese et al., 2014; van Zeventer et al., 2021). The most common mutation observed in CHIP is a C-to-T single-nucleotide substitution in the coding region, which occurs due to an age-dependent increase in the rate of spontaneous deamination of 5-methyl-cytosines at CpG loci (Alexandrov et al., 2013). Both *DNMT3A* and *TET2* mutations tend to be age-dependent (Buscarlet et al., 2017). *DNMT3A* and *TET2* mutations confer an advantage by enhancing self-renewal of stem and progenitor cells and inhibiting their differentiation (Challen and Goodell, 2020). The second most important risk factor for CHIP is history of chemotherapy and radiation. Therapy-related CH is frequently observed in patients with solid tumors (Coombs et al., 2017). Cancer therapy with radiation is associated with mutations in DNA damage response (DDR) genes (*TP53*, *PPM1D*, and *CHEK2*) (Bolton et al., 2020). It has been reported that DDR mutations are selected for due to their enhanced resistance to cytotoxic exposures (Challen and Goodell, 2020). Additionally, CHIP has been observed in patients who underwent autologous stem cell transplantation (ASCT) for multiple myeloma (MM) and lymphoma. Especially, *PPM1D* mutations were associated with inferior overall survival after ASCT for lymphoma (Gibson et al., 2017). Furthermore, smoking is reported to be strongly associated with CH, especially *ASXL1* mutation, in the United Kingdom Biobank cohort. The inflammatory environment induced by smoking might promote the outgrowth of *ASXL1*-mutant clones (Dawoud et al., 2020). Moreover, genetic factors have been reported to be risk factors for CHIP. Simultaneous analyses of germline and somatic whole-genome sequences revealed that mutations in the *TET2* distal enhancer in the germline lead to increased self-renewal of HSCs and CHIP (Bick et al., 2020). A whole-genome shotgun study showed that a germline variant of the telomerase reverse transcriptase (*TERT*) gene was associated with CHIP (Zink et al., 2017). Furthermore, other genome-wide analyses of United Kingdom Biobank participants revealed that germline mutations in DNA damage repair (*PARP1*, *ATM*, and *CHEK2*), HSC migration/homing (*CD164*), and myeloid oncogenesis (*SETBP1*) genes were associated with CH (Kar et al., 2022) (Table.1)

**TABLE 1:** The risk factors of CHIP and related references. We summarize the association between risk factors and related CH mutations in Table 1.

Risk factors	Related CH mutations	Association	References
**Age**	*DNMT3A* and *TET2*	an advantage by enhancing self-renewal of stem and progenitor cells and inhibiting their differentiation	Buscarlet et al. (2017), Challen and Goodell, (2020)
**Cancer therapy**	*TP53*, *PPM1D*, and *CHEK2*	mutations are selected for due to their enhanced resistance to cytotoxic exposures	Challen and Goodell (2020)
**ASCT**	*PPM1D*	inferior overall survival after ASCT for lymphoma	Gibson et al. (2017)
**smoking**	*ASXL1*	The inflammatory environment induced by smoking might promote the outgrowth of *ASXL1*-mutant clones	Dawoud et al. (2020)
**Genetic factors**		The germline variant of TERT, DNA damage repair (PARP1, ATM, and CHEK2), HSC migration/homing (CD164), and myeloid oncogenesis (SETBP1) genes were associated with CH	Zink et al. (2017), Kar et al. (2022)

CHIP, clonal hematopoiesis indeterminate potential; ASCT, autologous stem cell transplantation.

## 3 Positive feedback loop of inflammation and CHIP

HSCs harboring driver mutations in CHIP accelerate chronic inflammation. And, CHIP-associated mutations provide HSCs with competitive advantages under inflammatory conditions. Therefore, a positive feedback loop exists between chronic inflammation and CHIP, which leads to the worsening of inflammation and atherosclerosis. Recent studies have described inflammatory responses in macrophages or HSCs from murine models of *TET2*, *DNMT3A*, *ASXL1* and *JAK2* mutations which are the most common clonal hematopoiesis mutations (Balasubramani et al., 2015; Sano et al., 2018a; Fidler et al., 2021).

Hematopoietic *TET2* or *DNMT3A* disruption promotes both cardiac and renal fibrosis in hypertensive cardiac remodeling mice model via elevated expression of inflammatory cytokines such as IL-6 and C-C motif chemokine 5 (CCL5). *TET2* or *DNMT3A*-deficiency in myeloid cell lines promoted the inflammatory renponses (Sano et al., 2018a). Furthermore, in murine models of heart failure with *TET2* deficiency in hematopoietic cells, cardiac function worsened due to the elevation of IL-1β/*NLRP3* inflammasome, and the models responded better to IL-1β/*NLRP3* inflammasome inhibition (Sano et al., 2018b). Hypercholesterolemia-prone mice engrafted with bone marrow obtained from *TET2* knock-out mice had larger atherosclerotic lesions in the aorta than mice that received control bone marrow. In addition, macrophages from *TET2* knock-out mice secreted elevated levels of proinflammatory cytokines, such as IL1β and IL-6 (Jaiswal et al., 2017). Similarly, in dietary models of non-alcoholic steatohepatitis, mice transplanted with *TET2*-deficient hematopoietic cells demonstrated more severe liver inflammation and fibrosis due to elevated *NLRP3* inflammasome and downstream inflammatory cytokine expression in *TET2*-deficient macrophages (Wong et al., 2023). It has been shown that *DNMT3A* caused DNA hyper-methylation in the IL-6 promoter regions in synovial fibroblasts from osteoarthritis patients and suppressed the IL-6 expression (Yang et al., 2017). *TET2* has been reported to actively repress interleukin-6 (IL-6) transcription during inflammation resolution by recruiting histone deacetylase (HDAC) 2 in macrophages (Zhang et al., 2015). These papers suggested that *DNMT3A* or *TET2* deficiency promotes the inflammation.

CRISPR-Cas9-mediated sequential editing of human induced pluripotent stem cell-derived hematopoietic stem progenitor cells (HSPCs) resulting in *ASXL1*, *SRSF2*, and *NRAS* mutations activates innate immunity signaling pathways (Wang et al., 2021). *ASXL1*-MT knock-in mice show age-related expansion of phenotypic HSCs via overactivation of Akt/mTOR signaling through deubiquitination of Akt1 by the*ASXL1*-MT/Bap1 complex (Fujino et al., 2021). *DNMT3A* R878H knock-in mice, in which epigenetic regulation is disrupted, also develop hematologic malignancy through the overactivation of the mTOR pathway (Dai et al., 2017). The Akt/mTOR pathway is crucial for cell growth and survival (Porta et al., 2014), and its overactivation leads to mitochondrial activation, reactive oxygen species overproduction, and HSPC dysfunction (Fujino et al., 2021). This indicates that the mTOR pathway is a common pathology in CHIP. *JAK2* mutation, one of the most commonly observed mutations in CHIP, are associated with inflammation and atherosclerosis. *JAK2* ^*V617F*^(*JAK2* ^*VF*^) mutations increase the risk of developing premature coronary artery disease (Jaiswal et al., 2017). Increased proliferation and glycolytic metabolism of *JAK2* ^*VF*^ macrophages leads to DNA replication stress and *AIM2* inflammasome activation, thereby aggravating atherosclerosis (Fidler et al., 2021).

Clinical trials that used whole-genome sequencing data from several large cohorts showed that CHIP was strongly associated with epigenetic age acceleration (EAA), which is defined as the residual after regressing epigenetic clock age on chronological age, in several clocks, ranging from 1.31 years to 3.08 years (Nachun et al., 2021). Mutations in most CHIP genes, except DNA damage response genes, are associated with increases in several measures of age acceleration, and CHIP carriers are at a high risk of all-cause mortality and coronary heart disease (Nachun et al., 2021). In addition, EAA in adolescence is associated with obesity, inflammation (high sensitivity C-reactive protein and interferon-γ-inducible protein of 10 kDa (IP-10)), and future risk of cardiovascular diseases (Huang et al., 2019). IP-10 is a monocyte-derived proinflammatory chemokine that promotes the recruitment of lymphocytes and monocytes to site of inflammation. IP-10 is expressed in human atherosclerotic plaques (Profumo et al., 2010). Therefore, CHIP leads to EAA, systemic inflammation, and atherosclerosis. Aging and obesity are also reported to induce impaired hematopoietic regeneration by promoting expansion of the adipogenic lineage which produces an excess of dipeptidyl peptidase 4 (DPP4) (Ambrosi et al., 2017).

**FIGURE 2:**
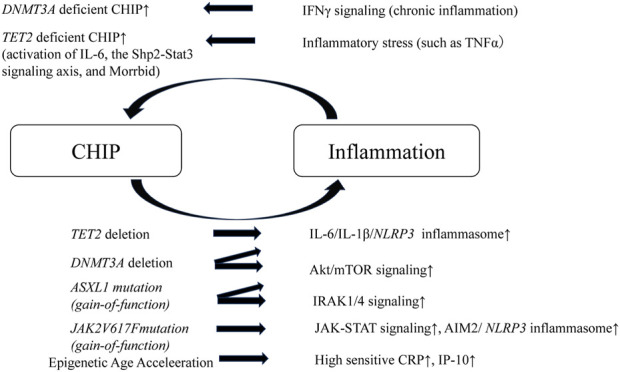
Schematic of positive feedback loop of inflammation and CHIP. A positive feedback loop exists between chronic inflammation and CHIP, which leads to the worsening of inflammation and atherosclerosis. *TET2* or *DNMT3A* deleted HSCs elevate IL-6/IL-1β/*NLRP3* inflammasome. *DNMT3A* deleted HSCs or *ASXL1* mutant HSCs (gain-of-function) overactivates Akt/mTOR signaling. *ASXL1* mutant HSCs also activates IRAK1/4 signaling. *JAK2* mutant HSCs and macrophages activate JAK-STAT signaling and enhance AIM2/*NLRP3* inflammasome. In addition, CHIP leads to pigenetic age acceleration, which results in elevation of high sensitive CRP and IP-10. In contrast, chronic inflammation drives CHIP. IFNγ under chronic inflammation drives *DNMT3A* deficient CHIP. *TET2*-deficient HSCs demonstrate a striking advantage in an inflammatory environment that contains TNFα and, *TET2* deficient mature myeloid cells and HSCs expand in response to inflammatory stress, which results in enhanced activation of IL-6, the Shp2-Stat3 signaling axis, and anti-apoptotic long non-coding RNA, Morrbid. CHIP, clonal hematopoietic indeterminate potential; HSCs, hematopoietic stem cells; IRAK1/4, IL-1 receptor-associated kinase 1 and 4; IP-10, interferon-γ-inducible protein of 10 kDa.

In contrast, chronic inflammation drives CH. For instance, IFNγ signaling induced during chronic infection drives *DNMT3A*-loss-of-function CH (Hormaechea-Agulla et al., 2021). Similarly, *TET2* deficient mature myeloid cells and HSCs expand in response to inflammatory stress, which results in enhanced activation of IL-6, the Shp2-Stat3 signaling axis, and anti-apoptotic long non-coding RNA, Morrbid (Cai et al., 2018). In addition, chronic inflammation induced by the activation of noncanonical NF-κB pathway contributes to a competitive advantage in MDS HSCs (Muto et al., 2020) (Figure 2).

## 4 CKD in CHIP and the possible mechanism of the kidney injury in CHIP

### 4.1 CHIP is associated with CKD

Several clinical trials have demonstrated the correlation between CHIP and CKD progression. In two independent cohorts of 87 and 85 adults, respectively, with an estimated glomerular filtration rate (eGFR) < 60 mL/min per 1.73 m^2^ (Kingston and CanPREDDICT cohorts), 25% of the individuals with advanced CKD had CHIP. Those with CHIP had a 2.2-fold greater risk of kidney failure over 5 years of follow-up and were more likely to have CKD complications, including anemia, than those without CHIP (Vlasschaert et al., 2022). In another study, CH was identified in 5449 (2.9%) eligible United Kingdom Biobank participants (n = 190,487), and it was found that CH was associated with CKD, which was defined by eGFR cystatin. Moreover, CH promotes adverse outcomes such as death, myocardial infarction, and stroke in CKD (Dawoud et al., 2022). In three community-based cohorts of the TOPMed Consortium (n = 12,004), the median baseline eGFR was 84 mL/min/1.73 m^2^, and the prevalence of CHIP was 6.6%, 9.0%, and 12.2% in persons aged 50–60, 60–70, and >70 years, respectively. A meta-analysis showed that CHIP was associated with a greater risk of 30% eGFR decline (17% [95%CI, 1%–36%] higher, *p* = 0.04). Additionally, the study revealed an association between CHIP and eGFR decline in three general population cohorts without known kidney disease (Kestenbaum et al., 2023). In contrast, a nested case-control study of the PROVALID study, which was a prospective cohort study including 4000 patients with T2DM in five European countries, showed that common risk factors (albuminuria, hemoglobin A1c, heart failure, and smoking) and microinflammation, but not CHIP, were associated with kidney function decline in T2DM (hazard ratio [HR] 1.06 [95% CI 0.57–1.96]) (Denicolo et al., 2022).

In experimental animal models, the recapitulation of CHIP has been shown to promote interstitial fibrosis and accumulation of macrophages in the kidneys (Sano et al., 2018a; Sano et al., 2018b). However, since these studies used a heart disease model (hypertensive cardiac remodeling or heart failure model), renal evaluation was a secondary endpoint. Therefore, the mechanisms underlying CHIP-related renal failure should be explored in animal models of kidney damage. Considering clinical and basic research, accumulation of mutated macrophages in the kidney might be the cause of renal fibrosis, glomerulosclerosis, and CKD, but the underlying mechanism remains unclear. Macrophages are the key regulators of tissue repair, regeneration, and fibrosis. Disturbances in macrophage function can lead to aberrant repair; deficient generation of anti-inflammatory macrophages; or failed crosstalk between macrophages and epithelial cells, endothelial cells, fibroblasts, and stem or tissue progenitor cells (Wynn and Vannella, 2016). Animal models have demonstrated that macrophages are major contributors to inflammatory responses in kidney injury and renal fibrosis (Huen and Cantley, 2017). Mutated macrophages generated from clonal expansion of myeloid cell line secrete cytokines such as IL-1β, IL-6, and TNFα and they might cause the injury of tubular epithelial cells or the activation of myofibroblast. In CHIP and kidney disease models, the pathology of impact of macrophages with CHIP on renal parenchymal cells should be verified.

### 4.2 A potential relationship between CHIP and monoclonal gammopathy of renal significance (MGRS)

A recent study showed that CHIP is associated with not only myeloid lineage malignancies but also lymphoid lineage ones, such as multiple myeloma (MM) and lymphoma (Husby et al., 2020; Wudhikarn et al., 2021). Recently, the kidney diseases caused by abnormal monoclonal antibodies generated by lymphoid lineage malignancies, which is called “monoclonal gammopathy of renal significance” (MGRS) has garnered attention among nephrologists and hematologists. MGRS is the disease concept that has recently been proposed (Leung et al., 2021). In this section, we propose the potential relationship between CHIP and MGRS.

Monoclonal gammopathy accompanied by lymphoid lineage malignancy is known as an important cause of kidney injury (Leung et al., 2021) Monoclonal gammopathy is defined as the presence of a monoclonal immunoglobulin in the plasma, urine, or both, which is often produced by clonal plasma cells, such as multiple myeloma (MM), and less commonly by B lymphocytes. Renal failure is a common complication of monoclonal gammopathy (Sy-Go et al., 2022). Until now, treatment for renal failure has not been recommended unless patients met the tumor burden criterion and had end-organ damage. Those patients had received careful monitoring as monoclonal gammopathy of undetermined significance (MGUS) because MGUS has risk of converting to malignancies such as MM, Waldenström macroglobulinaemia (WN), or chronic lymphocytic leukaemia (CLL) (Dimopoulos et al., 2009; Rajkumar et al., 2014; Strati and Shanafelt, 2015). However, it has been reported that there is B cell or plasma cell clonal disorder that does not fulfill the criteria for cancer yet produces a nephrotoxic monoclonal immunoglobulin that leads to kidney injury or disease, which was termed as MGRS (Leung et al., 2019).

Monoclonal immunoglobulins can cause kidney damage through various mechanisms that can be separated by the presence of a high or low tumor burden (Leung et al., 2021). Kidney injury from a high tumor burden is represented by light-chain cast nephropathy, which is characterized by monoclonal light chains that bind to Tamm-Horsfall protein through their variable domain to form obstructive casts (Sanders and Booker, 1992). However, in light chain cast nephropathy, high levels of serum free light chains are required, and almost all cases are accompanied by apparent hematological cancer such as multiple myeloma. Light-chain cast nephropathy is not considered an MGRS-related kidney lesion. MGRS is most commonly associated with low levels of monoclonal gammopathy. MGRS-associated renal lesions are initially separated by the presence or absence of monoclonal immunoglobulin deposits in kidney biopsy samples (Leung et al., 2019). Most MGRS-associated lesions are caused by the deposition of entire or parts of the monoclonal immunoglobulins or of various products of aggregation (Leung et al., 2019). The most common MGRS lesion is amyloid light chain (AL) amyloidosis. A small B-cell clone, most commonly a plasma cell clone, produces monoclonal light chains that exert organ toxicity and deposit in tissue in the form of amyloid fibrils (Palladini et al., 2020). Misfolding of a fragment of monoclonal immunoglobulin light chain occurs, resulting in the formation of toxic amyloid multimers and amyloid fibrils (Merlini and Bellotti, 2003). Renal involvement is very frequent in AL amyloidosis and can lead to the development of nephrotic syndrome followed by renal failure in some cases (Rysava, 2019). Other than AL amyloidosis, monoclonal immunoglobulin deposits related MGRS lesions include many diseases such as light-chain proximal tubulopathy (LCPT), immunotactoid glomerulonephritis, monoclonal fibrillary glomerulonephritis, cryoglobulinaemic glomerulonephritis, monoclonal immunoglobulin deposition disease (MIDD), and proliferative glomerulonephritis and monoclonal immunoglobulin deposits (PGNMID), which are relatively infrequent even among MGRS lesions (Leung et al., 2019). MGRS-associated lesions without deposits are C3 glomerulopathy with monoclonal gammopathy and thrombotic microangiopathy. Circulating monoclonal immunoglobulins are suspected to overactivate the alternative pathway, resulting in glomerular deposition of C3 or endothelial cell injury (Leung et al., 2021).

The diagnosis of MGRS can be established only by performing a kidney biopsy that either demonstrates the presence of monoclonal immunoglobulin deposits or infers their involvement with a circulating monoclonal immunoglobulin. Moreover, hematological evaluation such as peripheral blood flow cytometry and bone marrow biopsy is needed in order to detect a monoclonal immunoglobulin and predict treatment responses (Leung et al., 2019). Early diagnosis of MGRS is crucial, as it strongly impacts renal prognosis (Bridoux et al., 2015). Patients with MGRS require early effective treatment, such as clone-directed therapy, based on agents that were previously restricted to patients with overt hematologic cancers (Leung et al., 2021).

At the moment, the relationship between CHIP and MGRS has not verified. However, here is one clinical study which may indicate the relationship between CHIP and MGRS. CHIP mutations were observed in 21% among AL amyloidosis patients (Testa et al., 2022). In this study, CHIP was defined as the presence of *DNMT3A*, *TET2*, or *ASXL1* mutations in the peripheral blood or bone marrow (DTA-CH). Among CHIP mutations, *TET2* and *DNMT3A* were the most frequently mutated genes. And, DTA-CH did not predict worse overall survival (OS) or progression-free survival (PFS) in AL amyloidosis (Testa et al., 2022). However, this study included a relatively small sample size. And it did not include the data of proteinuria and renal function. Therefore, the clinical study focusing the relationship between CHIP and kidney function of MGRS patients is needed.

It has been reported that the type of CH mutations differs between myeloid and lymphoid lineage malignancies, and the type of mutation is highly predictive of patients being at risk of myeloid or lymphoid malignancies (Niroula et al., 2021). For example, it has been reported that the timing of CHIP related mutation occurrence differs in each gene. *DNMT3A* mutations occur in a multipotent HSC and affect both myeloid and lymphoid lineages. In contrast, *TET2* mutations occur in a more myeloid lineage committed HSCs and have myeloid bias (Buscarlet et al., 2017; Buscarlet et al., 2018).

**FIGURE 3:**
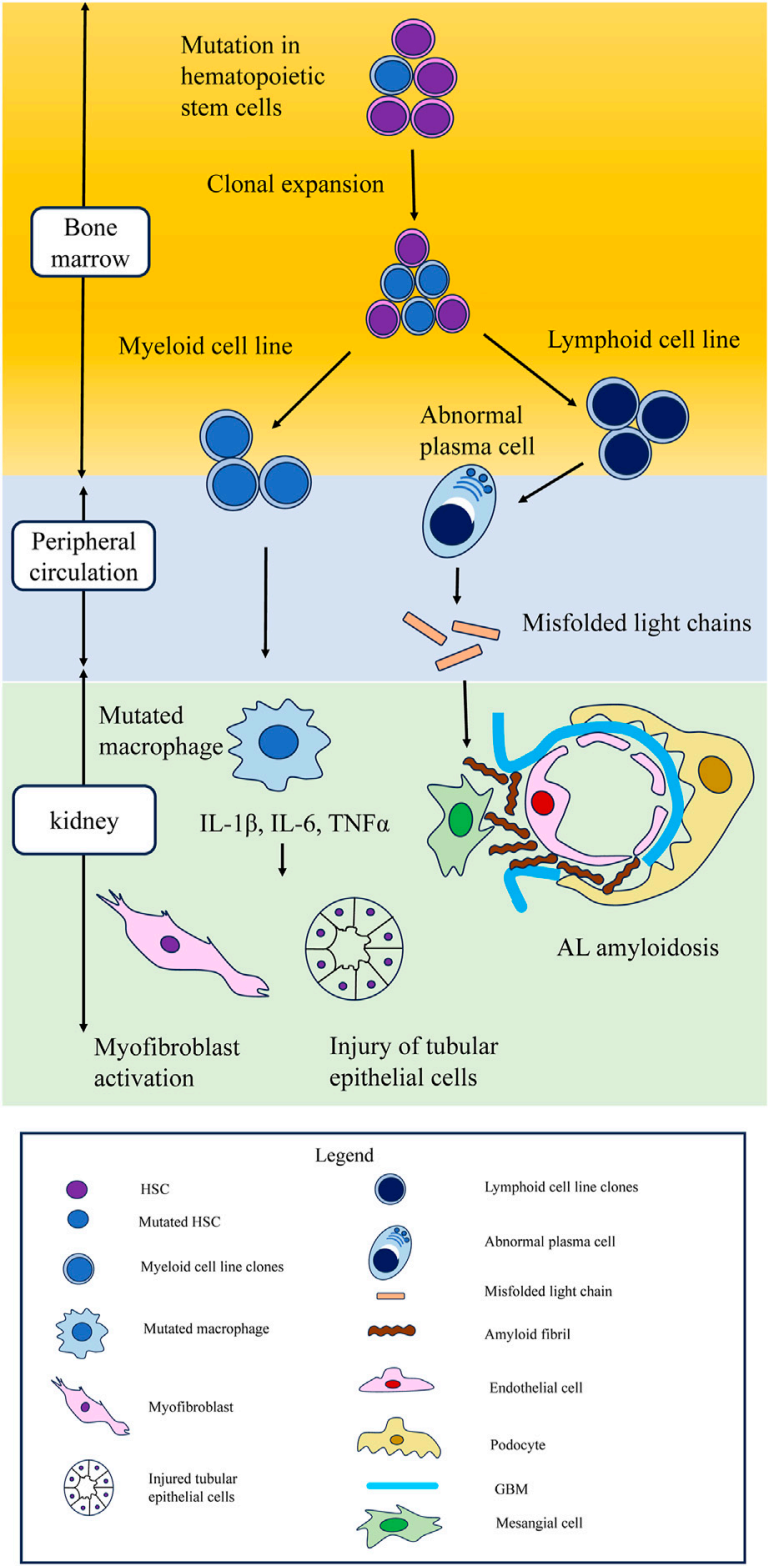
Schematic of hematopoietic stem cell mutations leading to clonal expansion in myeloid and lymphoid cell lines, leading to kidney damage. Possible mechanism of kidney injury in CHIP. Mutations in hematopoietic stem cells lead to clonal expansion in both myeloid and lymphoid cell lines. In myeloid cell line, cytokines such as IL-1β, IL-6, and TNFα secreted from mutated macrophages cause injury to tubular epithelial cells and myofibroblast activation. CHIP is also associated with lymphoid lineage malignancies, such as multiple myeloma and lymphoma. In lymphoid cell lines, aberrant monoclonal antibodies are related to monoclonal gammopathy of renal significance. In AL amyloidosis, which is the most typical MGRS lesion, abnormal plasma cells secrete misfolded light chains, resulting in the formation of toxic amyloid multimers and amyloid fibrils that deposit in the mesangium and the subepithelial space within glomeruli. CHIP, clonal hematopoietic indeterminate potential; MGRS, monoclonal gammopathy of renal significance; AL amyloidosis, amyloid light chain amyloidosis.

The type of CH mutations might influence the type of kidney injuries. In the first place, MGRS is difficult to diagnose and unfamiliar even among nephrologists. Many MGRS patients are thought to be undiagnosed among CKD patients. Further research on the association between CHIP and MGRS is warranted (Figure 3).

## 5 Epigenetic downregulation of Klotho in kidney and macrophage via inflammation

Kidney function steadily declines with age. Klotho, a renoprotective anti-aging protein secreted by the kidney, is involved in kidney aging process (Buchanan et al., 2020). Klotho has been identified as a central player in the aging-inflammation interface (Izquierdo et al., 2012). Enhanced inflammation is a common feature of CKD (Kadatane et al., 2023). Systemic or local renal inflammation decreases Klotho expression in the kidney (Izquierdo et al., 2012), and most kidney disorders are associated with a decline in circulating levels of Klotho mRNA and Klotho expression in the kidney (Wolf, 2014). As described previously, CHIP accelerates aging and induces systemic chronic inflammation, which leads to non-malignant diseases, including CKD. Thus, CHIP is regarded as one of the causes of decreased Klotho levels among the elderly.

In 1997, the Klotho gene was discovered when researchers observed that Klotho gene-disrupted mice exhibit a syndrome resembling human premature aging (Kuro-o et al., 1997). Klotho is mainly expressed in the kidneys, brain, parathyroid gland, and skeletal muscles. The distal convoluted tubule is the main site of Klotho expression in the kidneys (Kuro-o et al., 1997). Klotho exists in two general forms: as a transmembrane protein and a secretory form produced via alternative mRNA splicing of Klotho (Xu and Sun, 2015). Transmembrane Klotho is a co-receptor for fibroblast growth factor 23 (*FGF23*), which plays a critical role in the maintenance of phosphate and vitamin D homeostasis (Erben, 2016). In contrast, secreted Klotho protein regulates multiple growth factor signaling pathways, including insulin like growth-1, Wnt, and TGFβ1, and the activity of multiple ion channels and transporters (Izquierdo et al., 2012). Kidney Klotho expression responds to local and systemic inflammation, and epigenetic mechanisms, such as DNA methylation and histone modifications, and micro-RNA expression contribute to its downregulation (Ruiz-Andres et al., 2016).

DNA methylation suppresses gene expression and the subsequent protein translation. Inflammation-induced DNA hypermethylation resulted in Klotho promoter methylation, suppression of renal Klotho levels, and increased levels of the inflammatory marker CCL5 in animal models of renal interstitial fibrosis and human proximal tubular epithelial (HK-2) cells treated with CCL5 (Liu et al., 2022). Hypoxia and/or reoxygenation induced downregulation of Klotho expression has been observed in HK2 cells and renal tissues with ischemia-reperfusion injury (Zhao et al., 2022). In both the aforementioned studies, the DNA methyltransferase inhibitor 5-aza-2′-deoxycytidine (Aza) effectively reversed Klotho expression. Aza reduced Klotho promoter DNA methylation and exerted anti-apoptotic and anti-inflammatory effects by increasing Klotho expression in renal injury (Liu et al., 2022; Zhao et al., 2022). Methylation of the Klotho promoter has been observed in injured kidneys and peripheral blood leukocytes (PBLs). CCL5 upregulation concomitant with Klotho downregulation in the serum and DNA hypermethylation in PBLs has been observed in CKD patients (Liu et al., 2022). In another study, higher levels of Klotho promoter methylation were observed in the renal tissue and peripheral blood mononuclear cells (PBMC) of patients with CKD than in controls. The degree of Klotho promoter methylation in PBMCs was associated with the clinical and histological severity of CKD. PBMC Klotho promoter methylation level has been reported as a potential biomarker of renal Klotho promoter hypermethylation (Chen et al., 2013). The most frequently observed mutations in CH are loss of function mutations of *DNMT3A* and *TET2,* which are involved in DNA methylation and demethylation, respectively (Jaiswal, 2020). The relationship between *DNMT3A* or *TET2* mutations in CH and increase in Klotho promoter methylation levels in PBLs in CKD patients is unclear; therefore, further research is warranted.

Different combinations of histone modifications, such as methylation, acetylation, ubiquitination, and phosphorylation, regulate the chromatin structure and transcriptional status (Strahl and Allis, 2000). Histone acetylation relaxes the chromatin and facilitates transcription factor recruitment and transcription. Inflammatory cytokines, such as TNFα or TNF-like weak inducer of apoptosis (TWEAK), have been reported to promote the binding of NF-κB RelA (p65) to the Klotho promoter and thereby enhancing histone H3/H4 deacetylation of the murine Klotho promoter in cultured tubular cells, resulting in downregulation of Klotho expression. The histone deacetylase inhibitor trichostatin A prevents inflammatory cytokine induced Klotho downregulation (Moreno et al., 2011). The transcriptional activity of Klotho is also regulated by H3K9 modifications (Irifuku et al., 2016). In fibrotic kidney, TGFβ1, produced mainly by macrophages (Chevalier et al., 2009), increases the H3K9 methyltransferase G9a (Irifuku et al., 2016). Downregulation of Klotho levels, elevation of G9a, and monomethylation of H3K9 were observed in mouse models of unilateral ureteral obstruction and human kidney samples from patients with IgA nephropathy (Irifuku et al., 2016).

**FIGURE 4:**
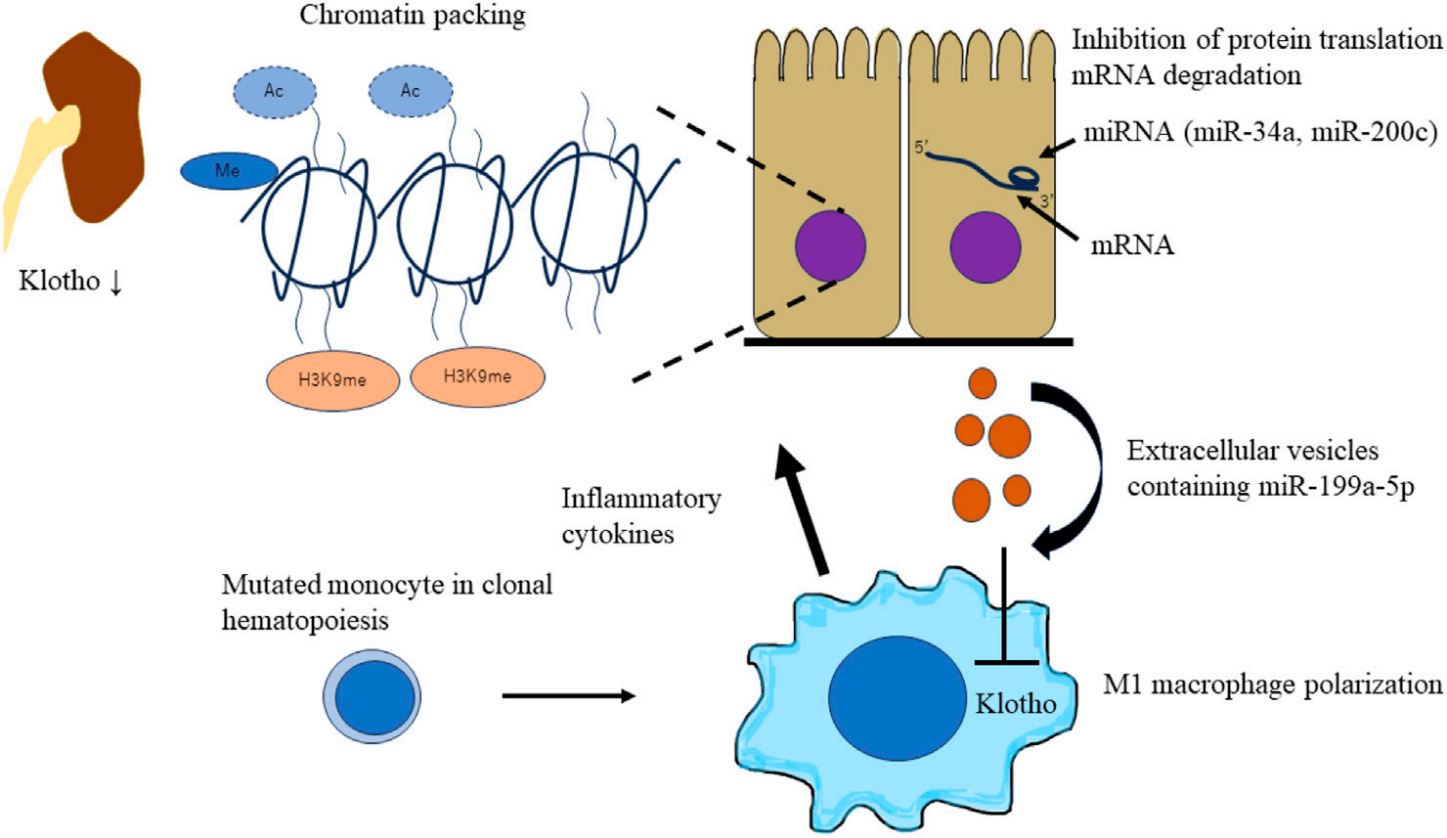
Schematic of epigenetic downregulation of kidney and macrophage Klotho levels by CHIP induced inflammation. CHIP-induced inflammation leads to epigenetic downregulation of kidney and macrophage Klotho levels. Inflammatory cytokines (TNFα or TWEAK) and TGFβ1 secreted by mutated macrophages induce chromatin packaging (Klotho promoter methylation, histone H3/H4 deacetylation, and H3K9 modification) in tubular epithelial cells. This leads to the decrease of Klotho levels in an injured kidney. miRNAs affect Klotho expression in renal cells and macrophages. miRNAs (miR-34a and miR-200c) aggravate kidney fibrosis by downregulating Klotho expression in tubular epithelial cells. Extracellular vesicles containing miRNAs (miR-100a-5p) from damaged tubular epithelial cells affect mutated macrophages. miRNAs suppress Klotho expression in macrophages and exacerbate the polarization of M1 macrophage phenotype. CHIP, clonal hematopoietic indeterminate potential; TWEAK, TNF-like weak inducer of apoptosis.

miRNAs are endogenous short non-coding RNAs of 22–25 base pairs that regulate gene expression through post-transcriptional repression of target mRNAs. miRNA binds to the 3′-untranslational region (UTR) of the target mRNA through base-pairing mechanism to suppress target gene expression by either mRNA degradation or inhibiting protein translation (Lu and Rothenberg, 2018). miRNAs regulate Klotho expression in renal diseases. miR-34a was reported to aggravate kidney fibrosis by downregulating Klotho expression in tubular epithelial cells (Liu et al., 2019). Oxidative stress-induced miR-200c binding to the Klotho mRNA 3′-UTR results in reduced Klotho expression in renal epithelial cells (Morii et al., 2019). miRNAs affect Klotho expression in renal cells and macrophages. Tissue macrophages are differentiated into M1 and M2 phenotypes on stimulation (Martinez et al., 2008). M1 macrophages (also called classically activated) are proinflammatory, which are activated by interferon gamma/lipopolysaccharide (IFN-γ/LPS). On the other hand, M2 macrophages (also called alternatively activated) are anti-inflammatory and promote wound healing and resolution of inflammation, which are activated by interleukin-4/interleukin-13 (IL-4/IL-13) (Huen and Cantley, 2017). miR-199a-5p from albumin-stimulated HK2 cell-derived extracellular vesicles (EVs) induces M1 polarization by suppressing Klotho in macrophages. In addition, Klotho polarizes M1 macrophages toward the M2 phenotype through Toll-like receptor 4 and ameliorates renal fibrosis in DM mouse models (Jia et al., 2019). In CKD with CHIP, mutated macrophages secrete inflammatory cytokines and damage renal cells. It has been speculated that miRNAs from damaged tubular epithelial cells affect mutated macrophages. miRNAs suppress Klotho expression in macrophages and exacerbate polarization of the M1 macrophage phenotype. A bidirectional worsening mechanism may exist between macrophages and tubular epithelial cells via Klotho and miRNAs (Figure 4).

## 6 The role of CHIP related genes in the kidney

The most frequently observed mutations in CHIP (*DNMT3A*, *TET2,* and *ASXL-1*) are of epigenetic regulators (Jaiswal et al., 2014). Epigenetic alterations are related to acute kidney injury-to-chronic kidney disease transition (Nangaku et al., 2017; Tanemoto et al., 2022; Tanemoto and Mimura, 2022), and the roles of *DNMT3A*, *TET2,* and *ASXL-1* in kidneys are reported as shown below.

DNA methylation involves DNA methyltransferase enzymes (DNMTs) such as *DNMT1*, *DNMT3A,* and *DNMT3B*. Maintenance of *DNMT*1 and *de novo* methyltransferases, *DNMT3A* and *DNMT3B,* are essential for mammalian development (Jin and Robertson, 2013). DNA methylation represses gene transcription and is associated with kidney injury (Tang and Zhuang, 2019). In studies on adenine-fed CKD mice, aberrant *DNMT1/DNMT3A* elevation, Klotho promoter hypermethylation, and Klotho suppression have been observed (Xia and Cao, 2021). *DNMT3A* and *DNMT3B* are responsible for the methylation of gene regulatory regions that act as enhancers during kidney development, but are decommissioned in adult mice. Although *DNMT3A* and *DNMT3B* knock-out mice displayed no obvious kidney abnormalities, they showed resistance to acute kidney injury (AKI). Human kidney disease risk loci were enriched in fetal regulatory regions (enhancers) that were decommissioned by *DNMT3A/3B* and were no longer active in adult kidneys. Genetic and epigenetic (*DNMT3A/3B*) factors may converge in the same genetic region, resulting in the development of kidney disease (Guan et al., 2020). Additionally, in renal fibrosis, the promoter region of *PTEN* is methylated and *PTEN* expression is downregulated. *DNMT3A* negatively regulates *PTEN* to activate the PI3K/AKT signaling pathway, induces epithelial-mesenchymal transition (EMT) in renal tubular epithelial cells, and aggravates renal fibrosis (Hu et al., 2022).

Ten eleven translocation (TET) methylcytosine dioxygenase family members catalyze the conversion of 5-methylcytosine to 5-hydroxymethylcytosine. The TET family of proteins, *TET1*, *TET2*, and *TET3* are involved in DNA demethylation. In addition, TET proteins play a role in regulating immunity and inflammation. In a study on *TET2* knock-out AKI mice model, it was observed that *TET2* exerted a renal-protective effect during AKI by regulating metabolic and inflammatory responses through the PPAR signaling pathway (Bao et al., 2021). Additionally, in mesangial cells, *TET2* activates TGFβ1 expression through demethylation of CpG islands in the TGFβ1 regulatory region under high-glucose conditions. Moreover, *TET2* plays an important role in the pathogenesis of diabetic nephropathy (Yang et al., 2018).

The additional sex comb-like (ASXL) family of proteins comprises chromatin factors that function in transcriptional activation and repression. It was originally identified in *Drosophila* as an enhancer of the trithorax and polycomb group proteins. Three Asx homologs have been identified in mammals: *ASXL1*, *ASXL2*, and *ASXL3*. Among them, *ASXL1* mutations are often found in a wide range of myeloid malignancies (Carbuccia et al., 2009) and Bohring-Opitz syndrome (Hoischen et al., 2011). *ASXL1* is essential for normal hematopoiesis and wild-type *ASXL1* epigenetically controls gene expression through its pivotal role in regulating the levels of H2AK119ub, H3K27me3, and H3K4me3 (Asada et al., 2019). In addition, wild-type *ASXL1* interacts with *AKT1,* and *ASXL1* deficiency in embryonic fibroblasts leads to p27-dependent cell cycle arrest, resulting in cellular senescence (Youn et al., 2017). Defects in kidney size and glomerular podocyte are observed in *ASXL1*-null mice; therefore, *ASXL1* is essential for normal podocyte development. *ASXL1* maintains podocyte structure via its association with Wilms’ tumor 1-interacting protein (WTIP) and the regulation of WT1 signaling during early kidney development (Moon et al., 2015). However, the role of *ASXL1* in kidney injury remains unclear.

## 7 Potential therapeutic approaches for CHIP and CHIP-related CKD

There are neither established therapies for CHIP nor definite criteria to determine eligibility for CHIP therapy yet. However, recent studies have supported the development of effective CHIP therapies, such as blocking the expansion of aberrant HSCs and inhibiting chronic inflammation. For example, in *TET2*-deficient mice, high-dose vitamin C reversed the aberrant self-renewal of HSPCs via *TET2* restoration (Cimmino et al., 2017). Additionally, since the mTOR pathway is a candidate pathway involved in CHIP pathology, Rapamycin, an mTOR inhibitor that effectively blocks the expansion of phenotypic HSCs in aged *ASXL1*-mutant knock-in mice (Fujino et al., 2021) can be a potential CHIP treatment.

**FIGURE 5:**
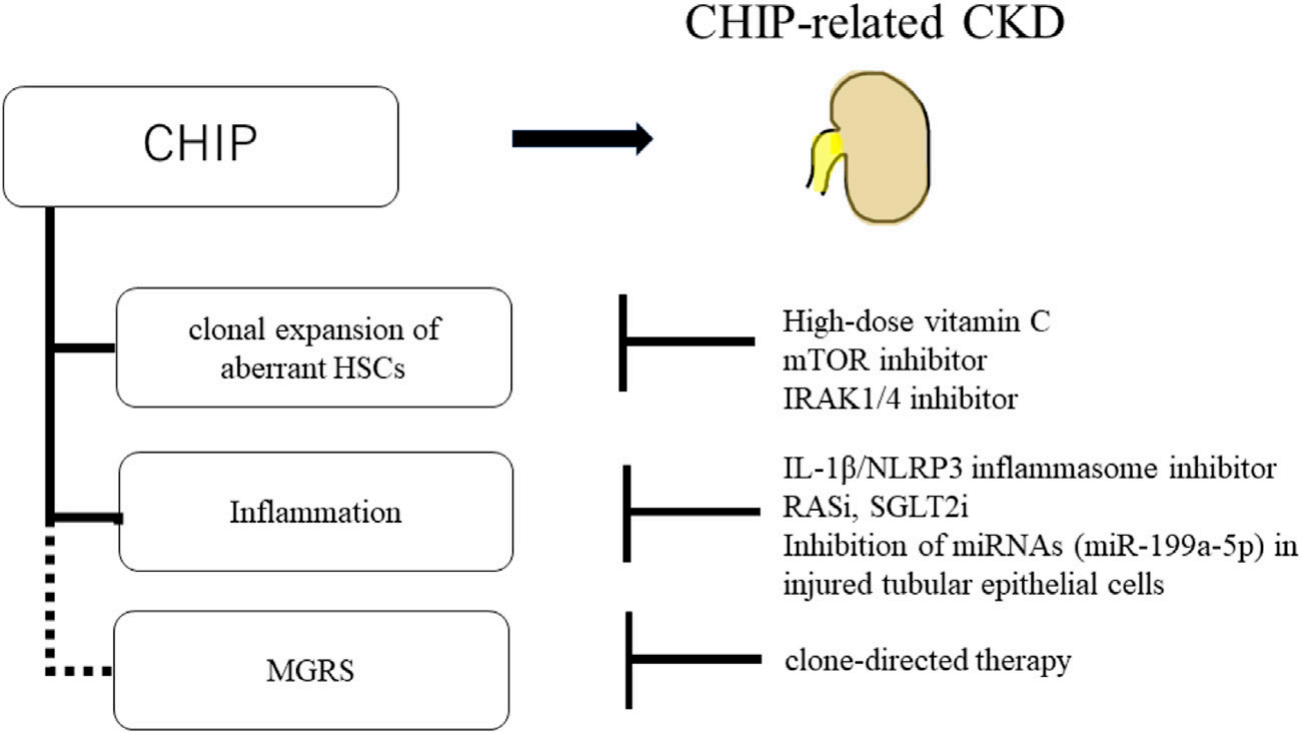
Schematic of potential therapeutic approaches for CHIP and CHIP-related CKD. High-dose vitamin C, mTOR inhibitor, and IRAK1/4 inhibitor prevent the clonal expansion of aberrant HSCs. And, IL-1β/*NLRP3* inflammasome inhibitor, RASi, SGLT2i, and inhibition of miRNAs (miR-199a-5p) in injured tubular epithelial cells suppress the inflammation in CHIP. There is potential relationship between CHIP and MGRS. If MGRS is diagnosed, clone-directed therapy is the treatment option.

Furthermore, the inhibition of inflammatory/innate signaling, such as the IRAK1/4 cascade, has been reported to prevent CHIP progression (Wang et al., 2021). In addition, anti-inflammatory drugs are effective against CHIP-related heart disease. In mouse models of heart failure with hematopoietic or myeloid *TET2* deficiency, IL-1β/*NLRP3* inflammasome inhibitor ameliorated heart failure (Sano et al., 2018b). CHIP-related CKD may be induced by mutated macrophages accumulating in the kidney, and anti-inflammatory drugs may be a treatment option for CKD. Renin-angiotensin system inhibitors (RASi) and sodium-glucose cotransporter-2 inhibitors (SGLT2i) are other potential candidates for CHIP treatment. RASi, angiotensin-converting enzyme inhibitors, and angiotensin receptor blockers are widely used to treat proteinuric CKD (Jafar et al., 2001). SGLT2i have been shown to delay CKD progression, regardless of the presence or absence of diabetes (Heerspink et al., 2020). Both RASi and SGLT2i have been reported to have anti-inflammatory effects (Cantero-Navarro et al., 2021; Winiarska et al., 2021), making them potential candidates for the treatment of CKD with CHIP. In addition, drugs targeting the epigenetic regulation of Klotho are a treatment option. EVs containing miRNAs (miR-199a-5p) from injured tubular epithelial cells have been shown to decrease Klotho expression and induce inflammation in macrophages (Jia et al., 2019). Therefore, inhibiting miRNAs in injured tubular epithelial cells may suppress the activation of inflammatory macrophages in patients with CKD with CHIP. Additionally, there is a potential relationship between CHIP and MGRS; therefore, if MGRS is diagnosed, clone-directed therapy may be required (Leung et al., 2021) (Figure 5).

## 8 Conclusion

Recent studies have revealed that CHIP is a risk factor of non-malignant diseases including CKD. There is a positive feedback loop between CHIP and inflammation. The possible mechanism of the kidney injury in CHIP is supposed to be due to the clonal expansion in both myeloid and lymphoid cell lines. Mutated macrophages in CHIP secrete cytokines which damage the organs including kidney. Inflammation leads to epigenetic downregulation of kidney and macrophage klotho which is anti-aging factor. And there is potential relationship between CHIP and MGRS which has emerged as an important factor of CKD among elderly patients. It is important to consider the CHIP related complications such as hematological malignancy, cardiovascular diseases and metabolic disorders in managing the elderly CKD patients.
